# Effects of Pre-Calving Body Condition and Different *post partum* Concentrate Feed Proportions on Immune-Associated and Hematological Parameters in Pluriparous Dairy Cows

**DOI:** 10.3390/ani10122251

**Published:** 2020-11-30

**Authors:** Katharina Bünemann, Jana Frahm, Susanne Kersten, Liane Hüther, Ulrich Meyer, Helga Sauerwein, Jürgen Hummel, Annette Zeyner, Sven Dänicke

**Affiliations:** 1 Institute of Animal Nutrition, Friedrich-Loeffler-Insitut (FLI), Federal Research Institute for Animal Health, 38116 Braunschweig, Germany; Katharina.Buenemann@fli.de (K.B.); Susanne.Kersten@fli.de (S.K.); Liane.Huether@fli.de (L.H.); ulrich.meyer@fli.de (U.M.); sven.daenicke@fli.de (S.D.); 2 Institute for Animal Science, Physiology, University of Bonn, Katzenburgweg 7–9, 53115 Bonn, Germany; sauerwein@uni-bonn.de; 3 Department of Animal Sciences, University of Göttingen, 37077 Göttingen, Germany; jhummel@gwdg.de; 4 Institute of Agricultural and Nutritional Sciences, Martin Luther University Halle-Wittenberg, 06120 Halle (Saale), Germany; annette.zeyner@landw.uni-halle.de

**Keywords:** dairy cow, pre-calving body condition score, lactation diet, immune system, hematology, peripheral blood monocular cells, inflammatory markers

## Abstract

**Simple Summary:**

Dairy cows have to cope with physiological, metabolic and endocrine challenges during the transition from late gestation to lactation. This period is characterized by a negative energy balance. Concentrate feed proportion of the rations is often increased to compensate the energy deficit. This can lead to acidotic conditions in the rumen, which might trigger the release of lipopolysaccharides from bacteria and result in inflammatory responses. An unfavorable pre-calving body condition is known to cause health problems after calving. Cows with a higher body condition (expressed as score, BCS, at a five-point scale) prior to calving might respond differently to either low or high concentrate feed proportions after calving with regard to hematological traits as indicators for possible interactions between nutritional status and immune system. For testing this hypothesis, cows were split into two classes (higher and adequate BCS) six weeks prior to calving according to their actual BCS and fed either a low- or high-energy-dense diet after calving. Neither BCS class nor dietary energy concentration influenced markedly hematological traits, including immune cell phenotypes, and inflammatory markers, such as haptoglobin. The overall covered individual BCS range and the *post partum* concentrate feed challenge did not overstress physiological adaptability.

**Abstract:**

The present study aimed at evaluating the influences of different concentrate feed proportions in the ration offered to dairy cows *post partum* with different body condition scores (BCS) before calving. Therefore, 60 pluriparous cows were divided 42 days before expected calving into two groups with a higher or an adequate BCS. After calving, both groups were further subdivided into a group fed a ration with either a low concentrate feed proportion (C, 35% at dry matter basis) or a high (60% at dry matter basis) one. It was hypothesized that different BCS would lead to different reactions concerning varying concentrate feed proportions. Isolated BCS effects were detected in the white blood profile only before calving. Neither low nor high concentrate feed proportions affected hematological, blood immune cell phenotypes and inflammatory markers consistently irrespective of BCS group. It was concluded, that the assessed BCS span covered a range in which the capability of cows to cope with different dietary *post partum* energy supply remained unchanged.

## 1. Introduction

Due to the transition from late gestation to lactation, the cow has to cope with physiological, metabolic and endocrine changes, which can be accompanied by high stress levels [1,2]. The individual body condition is known to influence the endocrine status, which in turn affects the immunological condition. Stress hormones, such as cortisol were proposed to change granulocyte (GR) count levels in blood [3]. Cortisol again, was shown to be negatively related to body condition [4]. However, several endogenous adaptions are physiological necessary, such as the degradation of tryptophan (Trp), which prevents a maternal immune reaction against the fetus [5]. All necessary adaptions are energy consuming processes at a time when the energy balance is already negative [6]. In prepartal cows over conditioning can increase the risk for complications at calving and thereafter [7]. Obesity was demonstrated to cause a high incidence of infections and alterations of immunity in both, humans and animals [8]. Cows with a higher pre-calving BCS exhibit a higher risk for accelerated lipolysis and consequently a more pronounced negative energy balance (NEB) *post partum* [7]. The suboptimal health status predispose for further health problems [9]. Haptoglobin (Hpt) is a major acute-phase-protein in cattle and used as indicator for an inflammatory response [10]. Hpt can also be regarded as a bovine adipokine and is therefore strongly related to body condition [11].

In experimental models, a low concentrate proportion of the ration is often used to increase lipolysis and enhance the risk of ketosis [12]. The energetic dilution of the ration would also increase the NEB [13]. This was confirmed in our previous study [14]. However, those experimental groups that were fed rations with lower concentrate feed proportions were not clearly prone to develop an excessive lipid mobilization. The non-esterified fatty acid (NEFA) concentrations of those groups exceeded the threshold for indicating an accelerated lipolysis. However, β-hydroxybutyrate (BHB) concentrations did not achieve the appropriate threshold [14] suggesting that NEFA utilization was not significantly compromised. In the present study, we aimed to investigate these relations more closely in the context of further immunological parameters.

Increasing concentrate proportions as an effort to counteract the NEB may also have some negative consequences for the cow. The change from a dry period diet to a lactation diet requires the adaptation of the rumen. Thus, an increasing concentrate proportion of the ration (C) can be considered as an additional challenge for the transition cow. Higher C may cause acidotic conditions in the rumen, which may result in subacute ruminal acidosis (SARA) [15,16]. These conditions lead to an increased lysis of Gram-negative bacteria, which release lipopolysaccharides (LPS) [17]. Additionally, the epithelium may be stressed and therefore more susceptible to injuries [18]. This would enable the translocation of LPS from the rumen into the blood stream [19] and the subsequently increased LPS transfer to the liver would trigger an acute-phase-response [20].

Several studies investigated the influence of *ante partum* BCS in combination with dry cow energy supply. These models often included different C during the dry period, in particular, to achieve an energy oversupply before calving and to stimulate *post partum* lipolysis [12,21,22]. However, to our knowledge, little is known about the possibly dynamic relation between pre-calving BCS variation covering a normal range and varying post-calving concentrate feeding regimen. Thereby, the pre-calving BCS of the animals in the current study ranged within a narrow range but separated cows into groups still significantly differing in BCS. Therefore, we aimed at evaluating the effects and interactions of different concentrate feed proportions in early lactating cows with different pre-calving body condition for that, we assessed hematological parameters, such as GR. It was hypothesized that if cows with higher BCS are characterized by a lower cortisol level an increased GR count level in blood would be expectable. Hüther et al. [23] described lower Kynurenine (Kyn): Trp-ratios in cows with higher BCS *post partum* compared to cows with lower BCS. Therefore, the question was, whether different concentrate proportions would dynamically change the BCS effect on that parameter. Another inflammation indicator assessed in the present study is Hpt, which was expected to be higher in higher conditioned cows due to its role as adipokine. Moreover, the CD14+ epitope is part of the LPS receptor complex and mainly present on the surface of monocytes. It belongs to the innate immune system, which can be triggered by endotoxins [24]. The crucial information we wanted to obtain by determining specific, immune-relevant surface markers such as CD4, CD8, CD14 and CD21 in peripheral blood leukocytes was to get an indication of immune-regulatory processes related to our experimental questioning.

## 2. Materials and Methods

The experiment was performed in compliance with the German legislation on animal protection (Animal Welfare Act) and approved by the Lower Saxony State Office for Consumer Protection and Food Safety (LAVES, Oldenburg, Germany) in consultation with an independent ethics committee (AZ 33.19-42502-04-15/1858). Milking performance and evaluation of nutritional status through ultrasonic-based estimation of the transition period-related dynamics of various adipose tissues were previously reported [14].

### 2.1. Experimental Design

The experiment included 60 pluriparous German Holstein cows and lasted over a time range from 42 days *ante partum* until 70 days in milk (DIM). Before parturition, all animals were either allocated to a group with a higher or an adequate BCS (BCS_H_, BCS_A_) based on the available BCS range of the herd. BCS was determined on a 5-point-scale according to Edmonson et al. [25]. Further criteria of classification were milk yield and milk composition of the previous lactation, as well as body weight and number of lactation. Until the day of calving, all animals received the same total mixed ration (TMR) with 80% silage (70% maize silage, 30% grass silage) and 20% concentrate on a dry matter (DM) basis. Supply of energy and nutrients was ensured based on the recommendations of the Society of Nutrition Physiology [26]. From the first day of lactation, all animals were supplied with a partial mixed ration (PMR) consisting of 48% maize silage, 20% grass silage and 32% concentrate on a DM basis.

After calving, cows were additionally assigned to a group with a concentrate feed proportion of 60% (increasing from 35–60% during the first three weeks after calving, C_60_) and a group with a concentrate feed proportion of 35% (C_35_). Hence, the following four groups were formed: BCS_H_/C_60_, *n* = 15, BCS_H_/C_35_, *n* = 15, BCS_A_/C_60_, *n* = 15, BCS_A_/C_35_, *n* = 15).

The components and the chemical compositions of the feedstuffs, as well as the experimental groups are described in detail elsewhere [14]. In brief, cows in the BCS_H_ group had an initial mean BCS of 3.83 (±0.41 standard deviation) while the BCS_A_ group started the experiment with a mean BCS of 3.10 (±0.38). Taking into account the fulfillment of the other criteria of classification, the initial BCS difference was 0.73 assessed as significant (*p* = 0.030). The average BCS during the experiment from week 1 to week 10 for the experimental groups were the following: BCS_H_/C_60_ = 3.44 (±0.72), BCS_H_/C_35_ = 3.23 (±0.85), BCS_A_/C_60_ = 2.70 (±0.67), BCS_A_/C_35_ = 2.66 (±0.53). A more detailed presentation of the BCS values over the course of the experiment was reported elsewhere [14].

### 2.2. Sample and Data Collection

Blood samples were taken at defined time points relative to calving (*ante partum*: −42 day, −14 day, −7 day, −3 day; *post partum*: 3 day, 7 day, 14 day, 21 day, 28 day, 42 day, 56 day, 70 day, with tolerated deviation of 2 days) after morning milking from a *Vena jugularis externa*.

EDTA blood samples were collected for flow cytometry and hematology. Serum samples were collected for Hpt and serum metabolites, such as Trp and Kyn. Blood for serum was centrifuged (Heraeus Varifuge 3.0R Heraeus, Osterode, Germany; 2123× *g*, 15 °C, 15 min) and stored at −80 °C until further analysis within 12 months.

#### 2.2.1. Hematology

For analyzing hematological parameters, EDTA-blood was used and a blood cell count was performed (Celltac MEK-6450, Nihon Kohden Corporation, Tokyo, Japan). Hematology included the white blood cell count with the count of total leukocytes (WBC), as well as cell counts of lymphocytes (LY), monocytes (MO), GR (comprising basophil and neutrophil GR) and eosinophils (EO), as well as their proportions of total WBC. Furthermore, the red blood cell profile was assessed, including the count of red blood cells (RBC), hemoglobin (HGB), hematocrit (HCT), the mean corpuscular volume (MCV), the mean corpuscular hemoglobin (MCH), the mean corpuscular hemoglobin concentration (MCHC) and the red cell distribution width (RDW).

#### 2.2.2. Flow Cytometry—Leukocytes Phenotyping

In order to assess the types of leukocytes, we performed flow cytometry using monoclonal antibodies. Thus, percentage of CD4+ cells represented T-helper lymphocytes, that of CD8+ represented cytotoxic T-cells, proportion of CD14+ reflected monocytes and finally, percentage of CD21+ represented B-cells. Blood-cell phenotyping was performed using whole blood. EDTA was used as anticoagulant for blood samples. Samples were incubated for 30 min at room temperature with monoclonal antibodies for CD4 and CD8 (mouse anti-bovine CD4: FITC; mouse anti-bovine CD8: PE; Bio-Rad, Hercules, CA, USA), as well as for CD14 and CD21 (mouse anti-bovine CD14: FITC; mouse anti-bovine CD21: PE; Bio-Rad) or their corresponding isotype controls (mouse IgG2a negative control: RPE and mouse IgG2b: FITC negative control, BioRad, Hercules, CA, USA).). To lyse the red blood cells, samples were subsequently incubated with a lysis buffer (BD Bioscience, San Jose, CA, USA) for 10 min at room temperature. Afterwards, samples were centrifuged, supernatant was removed and samples were resuspended in HEPES-buffered saline and then measured by FACS Canto II (BD Biosciences, San Jose, CA, USA).). By means of side- and forward-scattering properties, peripheral mononuclear blood cell (PBMC) subpopulations were identified. At least 10,000 PBMCs were counted and stored in list mode data files. The spillover of both fluorochromes (FITC, PE) was compensated using the BD FACS Diva^TM^ Software (BD Biosciences, San Jose, CA, USA).

#### 2.2.3. Haptoglobin and Serum Metabolites

Serum concentration of Hpt was measured from −42 d until 28 d, using an ELISA according to Hiss et al. [27]. Microplates were coated with bovine serum, blocked with casein and stored at 4 °C. The polyclonal antiserum against Hpt was added and incubated with samples, standard or controls for 2 hours at room temperature, after decanting the plates. The second enzyme-labeled antibody was added, after three time washing. Tetramethylbenzidin was used as chromogene. The optical density was determined at 450 nm. The limit of detection was set at 0.07 mg/mL. The intra- and inter-assay covariation was 9.99 and 11.67% respectively.

Trp and its degradation product Kyn were analyzed as described by Hüther et al. [23]. Fat extraction was performed by using hexane, and for protein precipitation, samples were mixed with ice cold ethanol. After centrifugation (20,800× *g*), the supernatant was quantitatively transferred into a flask and evaporated in a nitrogen stream at 40 °C. The residue was dissolved in aqueous mobile phase A and after filtration (amcro filter, PVDF, 0.45 µm) 20 µL were injected into a HPLC system (Shimadzu, Kyoto, Japan). Metabolites were separated by means of a reversed phase C18-column (Inertsil ODS-2, 150 × 3 mm i.d., 5 μm, Agilent, Böblingen, Germany), with a flow rate of 0.5 mL/min and a gradient elution. The mobile phase A consisted of 10 mM sodium 1-hexanesulfonate monohydrate, 0.5% (*v/v*) o-phosphoric acid and 0.5% (*v/v*) acetonitrile in ultrapure water; the mobile phase B consisted of 100% acetonitrile. For Trp and Kyn, the detection wavelengths were 278 nm and 360 nm, respectively. The intra- and inter-assay covariations were 3.1 and 6.3% (kynurenine) and 1.9 and 5.2% (tryptophan), respectively.

### 2.3. Statistical Analyses

**Figure 1:**
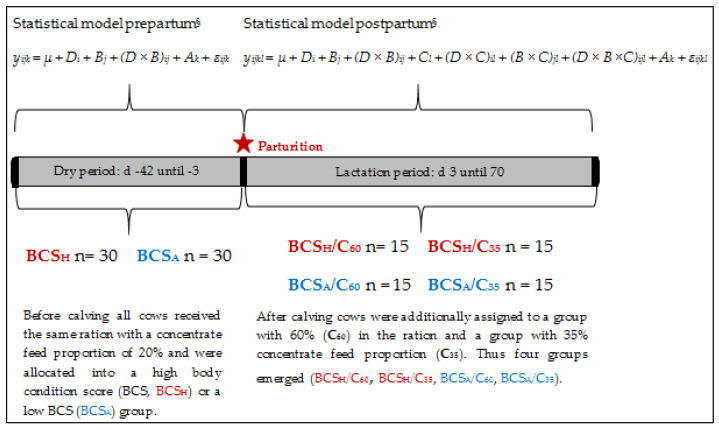
Statistical models. BCS: body condition scores; D_i_ = fixed effect of day (i = −42, …, −3/3, …, 70); B_j_ = fixed effect of BCS (j = BCS_H_, BCS_A_); A_k_ = effect of animal (k = 1,…,30,...,60); C_l_ = fixed effect of concentrate feed proportion (l = C_60_, C_35_); (D × B)_ij_, (D × C)_il_, (B × C)_jl_, (D × B ×C)_ijl_ = fixed effects of respective interactions, ε_ijkl_ = error.

For statistical analyses the software SAS was used (version 9.4; SAS Institute Inc., Cary, NC, USA). Variables of hematology and flow cytometry, as well as Hpt and the serum metabolites were evaluated using the MIXED procedure for repeated measurements with a compound symmetry structure [28]. The statistical analysis of the trail was divided into the time before and the time after parturition. BCS classification (BCS_H_, BCS_A_) and sampling day (−42, −14, −7, −3) were applied as fixed effects *ante partum*, as well as the interactions between them. The C effect (C_35_, C_60_) appeared *post partum* additionally to BCS classification and sampling day (3, 7, 14, 21, 28, 42, 56, 70). Each cow within treatment was considered a random effect. The sampling day was regarded to be a repeated measure. The statistical models are presented in Figure 1.

By assessing Pearson’s correlation coefficient, we examined relations between the individual BCS of each cow and selected parameters applying the statistical software TIBCO Statistica (Version 13.3, TIBCO Software Inc., Palo Alto, CA, USA). Furthermore, we performed linear regression analysis. The *p*-values ≤ 0.05 were declared to be statistically significant. Results are presented as LSMean ± Standard error of means (SEM) unless otherwise stated.

## 3. Results

### 3.1. Hematology

**Figure 2:**
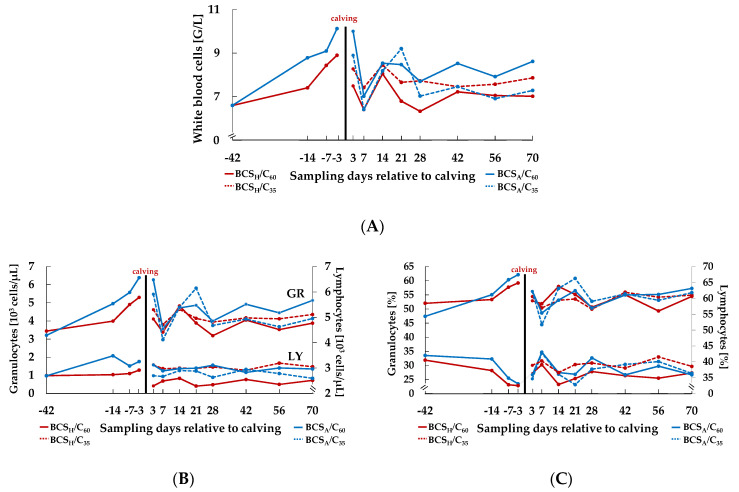
Characteristics of white blood cell counts (WBC, (**A**)), as well as counts (**B**) and percentages (**C**) of granulocytes (GR) and lymphocytes (LY) in the course of the experiment. BCS: body condition scores. *Ante partum*, cows were categorized in high body condition score (BCS, BCS_H_,) and adequate BCS (BCS_A_,). *Post partum*, the two groups were subdivided again, each into a group with a concentrate proportion of 60% (C_60,_ solid line) in the ration (increasing from 35–60% during the first three weeks after calving) and a group with a concentrate proportion of 35% (C_35_, dashed line) in the ration. Thus, four groups emerged: BCS_H_/C_60_ (*n* = 15), BCS_H_/C_35_ (*n* = 15), BCS_A_/C_60_ (*n* = 15), BCS_A_/C_35_ (*n* = 15).

The WBC (Figure 2A, Table 1) showed a gradual and continuous increase before and a decrease after calving. The BCS_A_ group developed *ante partum* higher WBC counts over time compared to the BCS_H_ group (effect Day × BCS *ante partum*, *p* = 0.047). *Post partum*, BCS_H_/C_60_ showed the lowest values, whereas BCS_A_/C_60_ developed the highest (effect BCS × C *post partum*, *p* = 0.046).

The GR counts (Figure 2B, Table 1) increased before calving, whereby BCS_A_ animals showed higher GR counts compared to BCS_H_ animals over time (effect Day × BCS *ante partum*, *p* = 0.006), the same applies for GR proportion (effect Day × BCS *ante partum, p* = 0.028). After calving, both GR counts (effect Day *post partum*, *p* < 0.001) and GR proportion (Figure 2C, Table 1, effect Day *post partum*, *p* < 0.001) changed over time with an initial decrease shortly after calving.

**Figure 3:**
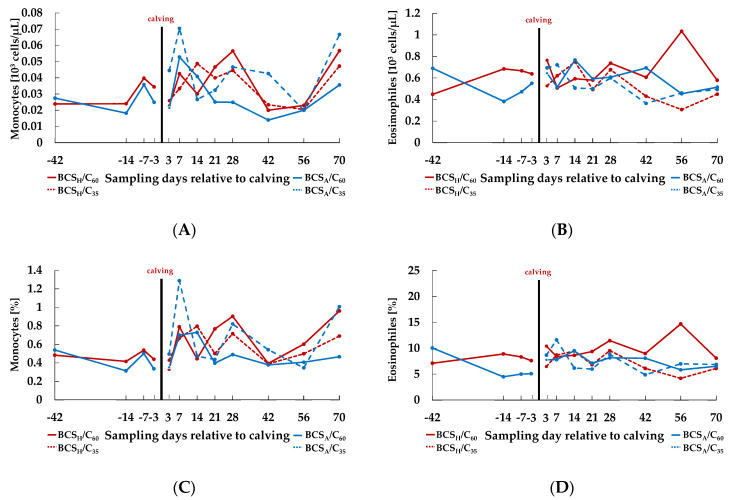
Characteristics of counts of monocytes (MO, (**A**)) and eosinophils (EO, (**B**)), as well as of percentages of MO (**C**) and EO (**D**) in the course of the experiment. *Ante partum*, cows were categorized in high body condition score (BCS, BCS_H_) and adequate BCS (BCS_A_). Post partum, the two groups were subdivided again, each into a group with a concentrate proportion of 60% (C_60,_ solid line) in the ration (increasing from 35–60% during the first three weeks after calving) and a group with a concentrate proportion of 35% (C_35_, dashed line) in the ration. Thus, four groups emerged: BCS_H_/C_60_ (*n* = 15), BCS_H_/C_35_ (*n* = 15), BCS_A_/C_60_ (*n* = 15), BCS_A_/C_35_ (*n* = 15).

The increase of percentage of GR *ante partum* was paralleled by a decrease of percentage of LY (Figure 2C, Table 1, effect Day *ante partum*, *p* < 0.001), whereas LY proportion increased *post partum* (effect Day *post partum*, *p* < 0.001). LY count (Figure 2B, Table 1) increased before and decreased after calving in all groups, except for BCS_H_/C_35_ animals, where values still increased after calving (effect Day *ante partum, p* = 0.004, effect Day × BCS × C *post partum, p* = 0.050). The MO count (Figure 3A, Table 2) and percentage (Figure 3C, Table 2) were not affected by treatment before calving. However, percentage of MO increased after calving (effect Day *post partum*, *p* = 0.001).

There was no treatment effect for EO count (Figure 3B, Table 2), neither before nor after calving. BCS_H_ animals developed *ante partum* higher percentages of EO (Figure 3D, Table 2) over time (effect Day × BCS *ante partum*, *p* = 0.017). After parturition, percentage of EO was also affected by BCS (effect BCS *post partum*, *p* = 0.047). The red blood profile is presented in the Appendix A.

### 3.2. Flow Cytometry—Leukocytes Phenotyping

**Figure 4:**
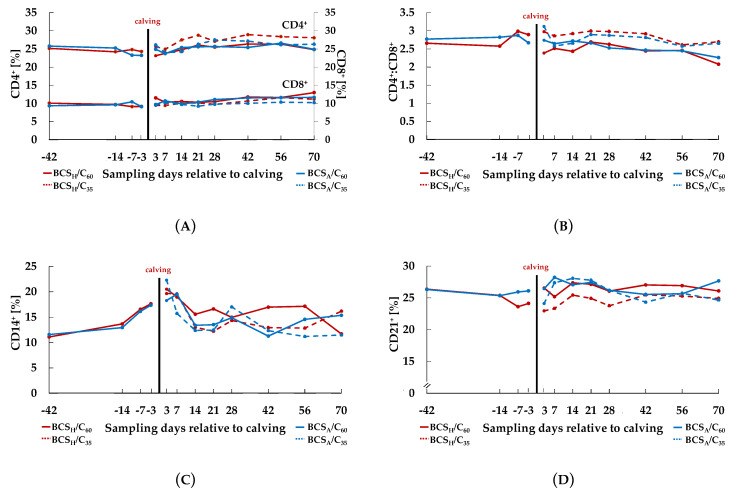
Percentage of CD4^+^ and CD8^+^ (**A**), CD4^+^:CD8^+^-ratio (**B**), as well as percentage of CD14^+^ (**C**) and CD21^+^ (**D**) in the course of the experiment. *Ante partum*, cows were categorized in high body condition score (BCS, BCS_H_) and adequate BCS (BCS_A_). *Post partum*, the two groups were subdivided again, each into a group with a concentrate proportion of 60% (C_60_, solid line) in the ration (increasing from 35–60% during the first three weeks after calving) and a group with a concentrate proportion of 35% (C_35_, dashed line) in the ration. Thus, four groups emerged: BCS_H_/C_60_ (*n* = 15), BCS_H_/C_35_ (*n* = 15), BCS_A_/C_60_ (*n* = 15), BCS_A_/C_35_ (*n* = 15).

In all experimental groups the percentage of CD4^+^ (Figure 4A, Table 3) decreased *ante partum* (effect Day *ante partum*, *p* = 0.013) and increased *post partum* (effect Day *post partum*, *p* < 0.001).

For the percentage of CD8^+^ (Figure 4A, Table 3) the same is true, although a time effect was observed after calving only (effect Day *post partum*, *p* < 0.001). After calving the ratio of CD4^+^:CD8^+^ (Figure 4B, Table 3) changed over time (effect Day *post partum*, *p* < 0.001). Values ranged between minimum = 2.08 (±0.25) and maximum = 3.12 (±0.25). The percentage of CD14^+^ (Figure 4C, Table 3) increased before calving (effect Day *ante partum*, *p* < 0.001), whereas *post partum* an initial decrease was noticed for all treatment groups followed by time-dependent fluctuations that were differently influenced by treatments (effect Day × BCS × C *post partum*, *p* = 0.005). We could not detect any significant effects on the percentage of CD21^+^ (Figure 4D, Table 3).

### 3.3. Haptoglobin and Serum Metabolites

**Figure 5:**
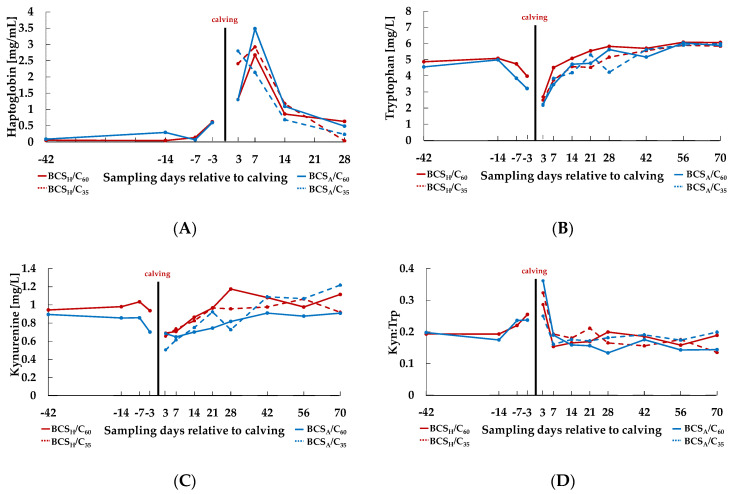
Blood concentrations of haptoglobin (Hpt, (**A**)), Tryptophan (Trp, (**B**)), Kynurenine (Kyn, **C**) and Kynurenine:Tryptophan-ratio (Kyn:Trp, **D**) in the course of the experiment. *Ante partum*, cows were categorized in high body condition score (BCS, BCS_H_) and adequate BCS (BCS_A_). *Post partum*, the two groups were subdivided again, each into a group with a concentrate proportion of 60% (C_60,_ solid line) in the ration (increasing from 35–60% during the first three weeks after calving) and a group with a concentrate proportion of 35% (C_35_, dashed line) in the ration. Thus, four groups emerged: BCS_H_/C_60_ (*n* = 15), BCS_H_/C_35_ (*n* = 15), BCS_A_/C_60_ (*n* = 15), BCS_A_/C_35_ (*n* = 15).

Hpt (Figure 5A, Table 4) was neither affected by BCS nor C, but increased within the first week after calving and decreased thereafter (effect Day *ante partum*, *p* = 0.011, effect Day *post partum*, *p* < 0.001).

Both, Trp (Figure 5B, Table 4) and its degradation product Kyn (Figure 5C, Table 4) decreased before and increased after calving. For both variables the BCS_H_ group exhibited higher values *ante partum* (effect BCS *ante partum*, *p* Trp = 0.046, effect BCS *ante partum*, *p* Kyn = 0.046). After calving, Trp, as well as Kyn increased (effect Day *post partum*, *p* Trp < 0.001, effect Day *post partum*, *p* Kyn < 0.001). The Kyn:Trp-ratio (Figure 5D, Table 4) showed the reverse picture, as it increased towards calving and decreased within 14 days after calving. For Kyn:Trp we detected a time effect (effect Day *ante partum*, *p* = 0.002) *ante partum*. Time and treatment affected Kyn:Trp in an interactive manner *post partum* (effect Day × BCS × C *post partum*, *p* = 0.040).

### 3.4. Correlations

Correlations were performed with the individual BCS of each cow and GR count, CD4^+^:CD8^+^-ratio, as well as Kyn:Trp-ratio and Hpt for the first two weeks after calving. None of the performed correlations revealed a significant relation.

## 4. Discussion

Although the BCS-difference of 0.73 was not great enough to induce significant effects on most of the parameters it was proven to be significant (*p* = 0.03) at the onset of the experiment. When setting up the experiment we considered the “normal” BCS range of our herd without artificial forcing for extremely low or high BCS animals through strong dietary manipulations. Therefore, we assigned cows from the given BCS range to groups with a higher and an adequate BCS. As these cow groups still differed significantly in mean BCS we hypothesized that BCS variation around a normal BCS would also influence metabolic and health traits significantly.

Cows with a higher BCS at calving are frequently considered being *post partum* more susceptible to metabolic disorders and vulnerable to infectious diseases through a compromised immune-responsiveness compared to cows with an adequate BCS [7]. As cows with higher BCS are less capable *post partum* to increase intake of DM and energy to appropriate levels, they suffer more from the *post partum* NEB than adequately conditioned cows. To investigate the influence of differences in BCS within an overall mean range often observable under practical feeding conditions, we grouped our herd into cows with a higher and an adequte BCS.

### 4.1. Hematology and Lymphocyte Subsets

The state of pregnancy is characterized by various changes in endocrine and metabolic alterations. Also, the transition from pregnancy to lactation is accompanied with an adaptive change in physiological, metabolic and immune status. The present study describes hematological changes of circulating cell populations during the transition period, reflected by a significant increase of WBC around parturition. Granulocytes as the major population of leukocytes were impacted markedly compared to lymphocytes which is in line with other studies [12,21]. A possible explanation for the increased GR counts towards calving is given by Burton et al. [3], who assumed an impaired trans-capillary-migration-capacity. Glucocorticoids induce a down-regulation of the expression of adhesion molecules on the surface of neutrophils, which results in a reduced infiltration in affected tissues. In addition, glucocorticoids, such as cortisol, stimulate the release of immature neutrophil granulocytes from the bone marrow [3]. Around calving, cows are exposed to high stress levels, due to exogenous and endogenous changes, such as the adaption from a dry period diet to a lactation diet or the transition from late gestation to lactation [2,21,29]. These marked endocrine changes might explain the observed time course in granulocyte counts in particular and in white blood profile in general. Endo et al. [4] showed a negative correlation between BCS and hair cortisol concentration. If cows with a higher BCS are characterized by a lower cortisol level an increased granulocyte count in blood would be expectable which, however, was not observed in the present experiment. Obviously, the BCS in the BCS_H_ groups was not high enough to induce significant differences in cortisol levels compared to the BCS_A_ groups.

Based on this assumption and the known influence of cortisol on release of granulocytes, it seems comprehensible that GR count increased more pronounced in BCS_A_ groups before calving in the present study. Burton et al. [3] proposed that the surge of cortisol could also be fetus-derived and that it induced adaptive changes of the neutrophil system, which resulted in the favoring of tissue remodeling instead of antibacterial defense. This, in turn, could increase the susceptibility to diseases [2]. Chapwanya et al. [30] demonstrated endometrial infiltration of leukocytes, mainly neutrophil aggregates and elevated expression of pro-inflammatory genes in uteri of *post partum* cows, indicating endometrial inflammatory processes, which explain the *post partum* decrease of GR counts in the present study. The authors described these inflammations as necessary events and beneficial for normal endometrial involution and bacterial clearance.

A higher BCS *ante partum* often leads to complications at calving [7,8] with consequences for *post partum* susceptibility to metabolic disorders and infectious diseases. Even though the BCS groups did not differ concerning WBC and GR count at the start of the experiment (−42 d), the increase of both parameters was less pronounced in the BCS_H_ groups *ante partum* in the present study without consequences for the dynamics of these cell types *post partum*. Although for lymphocyte counts similar relations were observed *ante partum*, this cell type fluctuated at a lower level when cows with a higher BCS were supplied with more energy (BCS_H_C_60_) *post partum*. In contrast, Eger et al. [31] denied a relation between body condition and lymphocytes, when grouping the experimental cows in lower BCS (mean 2.77) and higher BCS (mean 3.73). The BCS difference of the experimental groups in the present study was even smaller and might not have been large enough to trigger marked effects on lymphocytes.

In accordance with this result, the pre-calving BCS itself did not affect lymphocyte subsets, such as CD4^+^ and CD8^+^ in the current investigation. The percentage of CD4^+^, as well as that of CD8^+^ increased after calving, which is in accordance with previous reports [21,32].

The CD4^+^:CD8^+^-ratio, which was neither affected by treatment, remained within the range indicating a balanced immune homeostasis, as described in previous studies [33,34]. The correlation between BCS and CD4^+^:CD8^+^-ratio was performed to examine the relation on an individual basis. The results support the idea of the nonexistent relation between the body condition and the CD4^+^:CD8^+^-ratio at least within the rather small depicted BCS range of the present study.

In the present study, CD14^+^ cells decreased in all groups after calving, independent of experimental treatment. Endometrial inflammatory processes and uterine bacterial infections were observed in *post partum* dairy cows. These infections are associated with LPS, which translocate into the uterus [30,35]. Not related to treatment 33% of the cows in the BCS_H_/C_60_ group, 40% of the animals in both the BCS_H_/C_35_ and BCS_A_/C_35_ group and 53% of the cows in the BCS_A_/C_60_ group developed production diseases, such as mastitis and metritis. This results in migration of monocytes, which in turn explains the decrease of peripheral CD14^+^ cells after calving in the present study. The interaction between time, BCS and C in the current study suggests that an enhanced C in combination with a higher BCS triggered a higher CD14^+^ cell proportion.

### 4.2. Haptoglobin and Kynurenine:Tryptophan-Ratio

The results of this experiment indicate that a BCS effect on mobilization was only detectable in individual fat depots [14]. It was not verifiable that higher conditioned cows have a higher potential to mobilize body fat in general. Moreover, compared to the hepatic production the Hpt synthesis of the subcutaneous and visceral adipose tissue constitutes only 0.02% of the overall synthesis [11]. Based on these facts the unaltered Hpt levels in higher BCS cows of the present study might be explained.

Other studies already indicated that rations differing in C did not influence Hpt and even when drastically increasing C, circulating Hpt remained unaltered [13,36]. Moreover, Drong et al. [22] demonstrated, that Hpt was even unaffected by different BCS in combination with different dietary compositions. Furthermore, the regulation of acute-phase-proteins is described as a function of several parameters, which interact in a complex manner [37]. The individual cow factor explains 22% of the Hpt variation [38].

The Kyn:Trp-ratio provides information about the activity of indoleamine-2,3-dioxygenase, which is activated during inflammations or infections and also suggested to regulate the implantation of the embryo. Due to its degradation via the kynurenine-pathway it leads to a decrease of Trp during gestation. The Trp degradation prevents a maternal immune reaction against the fetus [5,39]. Therefore, the increase of the Kyn:Trp-ratio towards calving observed in the present study is physiological necessary [5,40,41]. Hüther et al. [23] described lower Kyn:Trp-ratios in cows with higher BCS *post partum* compared to cows with adequate BCS. Although we observed a three-way interaction between time, concentrate feed proportion and BCS class *post partum*, a clear effect of BCS was not detected. The relation between BCS and several immune parameters is non-linear and therefore, the impacts are difficult to predict [7,13]. This becomes also apparent in the missing significance for the correlation between BCS and Kyn:Trp-ratio. However, in the afore mentioned study, dry cow nutrition differed concerning C, whereby different diets did not impact the Kyn:Trp-ratio. The mentioned interactions in the present study suggest that different energy levels after calving have greater influence on several parameters than varying dry cow nutrition.

## 5. Conclusions

All assessed immunological variables changed over time and clearly reflect the transition period as challenging time for dairy cows. It was hypothesized that cows differing in BCS would respond differently to rations varying in concentrate feed proportion *post partum*. Based on the investigated hematological parameters, blood immune cell phenotypes, and inflammatory markers it can be concluded that the given BCS variation in the present study covered a range in which physiological adaptability was not overstretched.

## Figures and Tables

**Table 1:** Model corresponding to Figure 2.

		*p*-Values (before Calving) *					
	**Day**	**BCS**	**Day × BCS**				
A WBC	<0.001	0.063	0.047				
B GR count	<0.001	0.029	0.006				
B LY count	0.004	0.084	0.054				
C GR%	<0.001	0.679	0.028				
C LY%	<0.001	0.225	0.637				
		***p*** -Values (after Calving) *					
	**Day**	**BCS**	**Day × BCS**	**C**	**Day × C**	**BCS × C**	**Day × BCS × C**
A WBC	<0.001	0.124	0.091	0.811	0.761	0.046	0.905
B GR count	<0.001	0.084	0.099	0.988	0.927	0.172	0.749
B LY count	0.968	0.421	0.815	0.243	0.899	0.018	0.050
C GR%	<0.001	0.415	0.388	0.959	0.965	0.909	0.552
C LY%	<0.001	0.888	0.436	0.376	0.855	0.266	0.848

* before calving: −42 day, −14 day, −7 day, −3 day; after calving: 3 day, 7 day, 14 day, 21 day, 28 day, 42 day, 56 day, 70 day (with tolerated deviation of 2 days). WBC: blood cell counts; BCS: body condition scores; GR: granulocytes; and LY: lymphocytes.

**Table 2:** Model corresponding to Figure 3.

	***p*** -Values (before Calving) *						
	**Day**	**BCS**	**Day × BCS**				
A MO count	0.465	0.677	0.921				
B EO count	0.975	0.392	0.051				
C MO%	0.280	0.606	0.786				
D EO%	0.252	0.145	0.017				
	***p*** -Values (after Calving) *						
	**Day**	**BCS**	**Day × BCS**	**C**	**Day × C**	**BCS × C**	**Day × BCS × C**
A MO count	0.011	0.988	0.686	0.520	0.964	0.210	0.824
B EO count	0.467	0.164	0.663	0.070	0.152	0.408	0.135
C MO%	0.001	0.552	0.692	0.473	0.939	0.089	0.229
D EO%	0.435	0.047	0.741	0.079	0.311	0.062	0.201

* before calving: −42 day, −14 day, −7 day, −3 day; after calving: 3 day, 7 day, 14 day, 21 day, 28 day, 42 day, 56 day, 70 day (with tolerated deviation of 2 days). MO: monocytes; EO: eosinophils.

**Table 3:** Model corresponding to Figure 4.

	***p*** -Values (before Calving) *						
	**Day**	**BCS**	**Day × BCS**				
A CD4^+^	0.013	0.825	0.096				
A CD8^+^	0.902	0.902	0.585				
B CD4^+^:CD8^+^	0.204	0.984	0.313				
C CD14^+^	<0.001	0.780	0.863				
D CD21^+^	0.155	0.490	0.298				
	***p*** -Values (after Calving) *						
	**Day**	**BCS**	**Day × BCS**	**C**	**Day × C**	**BCS × C**	**Day × BCS × C**
A CD4^+^	<0.001	0.458	0.494	0.090	0.834	0.420	0.885
A CD8^+^	<0.001	0.509	0.603	0.193	0.834	0.898	0.615
B CD4^+^:CD8^+^	<0.001	0.968	0.869	0.128	0.654	0.547	0.939
C CD14^+^	<0.001	0.250	0.321	0.212	0.074	0.640	0.005
D CD21^+^	0.314	0.409	0.242	0.250	0.912	0.541	0.934

* before calving: −42 day, −14 day, −7 day, −3 day; after calving: 3 day, 7 day, 14 day, 21 day, 28 day, 42 day, 56 day, 70 day (with tolerated deviation of 2 days).

**Table 4:** Model corresponding to Figure 5.

	***p*** -Values (before Calving) *						
	**Day**	**BCS**	**Day × BCS**				
A Hpt	0.011	0.817	0.972				
B Trp	<0.001	0.046	0.357				
C Kyn	0.294	0.046	0.414				
D Kyn:Trp	0.002	0.836	0.742				
	***p*** -Values (after Calving) *						
	**Day**	**BCS**	**Day × BCS**	**C**	**Day × C**	**BCS × C**	**Day × BCS × C**
A Hpt	<0.001	0.958	0.988	0.884	0.146	0.687	0.642
B Trp	<0.001	0.211	0.887	0.161	0.608	0.329	0.299
C Kyn	<0.001	0.296	0.606	0.675	0.125	0.380	0.664
D Kyn:Trp	<0.001	0.806	0.969	0.503	0.329	0.972	0.040

* before calving: −42 day, −14 day, −7 day, −3 day; after calving: 3 day, 7 day, 14 day, 21 day, 28 day, 42 day, 56 day, 70 day (with tolerated deviation of 2 days). Hpt: Haptoglobin; Trp: Tryptophan; Kyn: Kynurenine; Kyn:Trp: Kynurenine:Tryptophan-ratio

**Figure A1:**
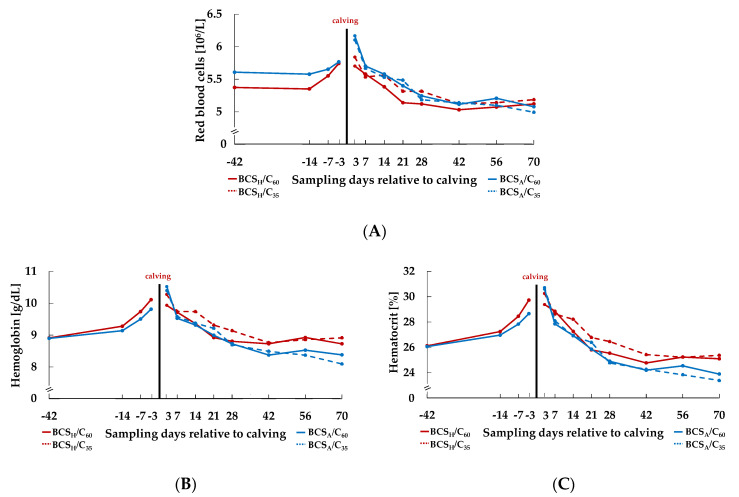
Characteristics of red blood cell counts (**A**)**,** Hemoglobin (**B**) and Hematocrit (**C**) in the course of the experiment. *Ante partum*, cows were categorized in high body condition score (BCS, BCS_H_) and adequate BCS (BCS_A_). *Post partum*, the two groups were subdivided again, each into a group with a concentrate proportion of 60% (C_60,_ solid line) in the ration (increasing from 35–60% during the first three weeks after calving) and a group with a concentrate proportion of 35% (C_35_, dashed line) in the ration. Thus, four groups emerged: BCS_H_/C_60_ (*n* = 15), BCS_H_/C_35_ (n = 15), BCS_A_/C_60_ (*n* = 15), BCS_A_/C_35_ (*n* = 15). ^*^ before calving: −42 day, −14 day, −7 day, −3 day; after calving: 3 day, 7 day, 14 day, 21 day, 28 day, 42 day, 56 day, 70 day (with tolerated deviation of 2 days).

## Appendix A

RBC (Figure A1A, Table A1) increased towards calving in BCS_A_ and BCS_H_ groups (effect Day *ante partum*, *p* = 0.003).

**Table A1:** Model corresponding to Figure A1.

	***p*** **-Values (before Calving) ***						
	**Day**	**BCS**	**Day × BCS**				
A RBC	0.003	0.209	0.468				
B HGB	<0.001	0.348	0.737				
D HCT	<0.001	0.310	0.642				
	***p*** **-Values (after Calving) ***						
	**Day**	**BCS**	**Day × BCS**	**C**	**Day × C**	**BCS × C**	**Day × BCS × C**
A RBC	<0.001	0.571	<0.001	0.520	0.146	0.629	0.797
B HGB	<0.001	0.117	<0.001	0.354	0.608	0.619	0.658
D HCT	<0.001	0.074	<0.001	0.512	0.329	0.681	0.702

* after calving, BCS_A_ groups developed a higher peak at d 3 (effect Day × BCS *post partum*, *p* < 0.001). RBC: red blood cell counts; HGB: Hemoglobin; HCT: Hematocrit.

Both, HGB (Figure A1B, Table A1) and HCT (Figure A1C, Table A1) increased before calving (effect Day *ante partum*, *p* HGB < 0.001, effect Day *ante partum*, *p* HCT < 0.001). After calving, HGB decreased more pronouncedly in BCS_H_ groups compared to BCS_A_ groups (effect Day × BCS *post partum*, *p* HGB < 0.001). The same is true for HCT (effect Day × BCS *post partum*, *p* HCT = 0.001). Both parameters peaked around calving.

MCV (Table A2) increased from day −42 to day −14 and remained relatively constant afterwards in all groups (effect Day *ante partum*, *p* < 0.001). Day and C affected MCV *post partum* in an interactive manner (effect Day × C *post partum*, *p* < 0.001).

The same is true for MCH (Table A2, effect Day *ante partum*, *p* < 0.001, effect Day × C *post partum*, *p* = 0.035). Additionally, the BCS_H_ groups had *post partum* higher values for MCH compared to BCS_A_ groups over time (effect Day x BCS *post partum*, *p* = 0.029). For MCHC (Table A2) no effect was found *ante partum*, whereas Day, BCS and C influenced MCHC *post partum* in an interactive manner (effect Day × BCS × C *post partum*, *p* < 0.001). RDW (Table A2) increased *ante partum*, and decreased *post partum*, in all groups (effect Day *ante partum*, *p* < 0.001, effect Day *post partum*, *p* < 0.001).

**Table A2:** *Ante partum* effects of body condition score (BCS) and Days of experiment (Day), as well as *post partum* effects of BCS, concentrate feed proportion in the diet (C) and Day on parameters of the red cell indices.

**Item ^+^**	**Group ^§^**	**Day of Experiment—*ante partum***								**SEM ^#^**	***p*** **-Value**						
		**−42**	**−14**	**−7**	**−3**						**Day**	**BCS**	**Day × BCS**				
MCV ^$^, fL	BCS_H_	48.7	51.2	51.4	51.8					0.7	<0.001	0.055	0.818				
	BCS_A_	46.6	49.1	49.7	49.8												
MCH ^¥^, pg	BCS_H_	16.6	17.4	17.6	17.6					0.3	<0.001	0.052	0.496				
	BCS_A_	15.9	16.6	16.9	17.0												
MCHC ^ǁ^, g/dL	BCS_H_	34.1	34.0	34.3	34.0					0.1	0.528	0.829	0.783				
	BCS_A_	34.1	33.9	34.1	34.2												
RDW ^◊^,%	BCS_H_	17.54	18.87	18.46	18.38					0.55	<0.001	0.093	0.835				
	BCS_A_	18.27	20.17	19.66	19.43												
**Item ^+^**	**Group ^$^**	**Day of Experiment—*post partum***								**SEM ^#^**	***p*** **-Value**						
		**3**	**7**	**14**	**21**	**28**	**42**	**56**	**70**		**Day**	**BCS**	**Day × BCS**	**C**	**Day × C**	**BCS × C**	**Day × BCS × C**
MCV ^$^, fL	BCS_H_/C_60_	51.6	51.8	50.7	50.3	50.0	49.3	49.9	49.2	0.9	<0.001	0.018	0.665	0.944	<0.001	0.883	0.454
	BCS_H_/C_35_	52.1	52.1	51.0	50.6	50.1	49.8	49.3	49.1								
	BCS_A_/C_60_	49.5	49.0	48.4	48.0	47.7	47.5	47.2	47.1								
	BCS_A_/C_35_	50.5	49.8	48.8	48.2	48.0	47.4	46.9	47.0								
MCH ^¥^, pg	BCS_H_/C_60_	17.5	17.5	17.4	17.4	17.2	17.4	17.7	17.1	0.3	<0.001	0.022	0.029	0.879	0.035	0.952	0.174
	BCS_H_/C_35_	17.7	17.7	17.6	17.6	17.3	17.2	17.3	17.3								
	BCS_A_/C_60_	17.0	16.8	16.7	16.7	16.7	16.4	16.4	16.5								
	BCS_A_/C_35_	17.1	16.9	17.0	16.8	16.9	16.6	16.5	16.3								
MCHC ^ǁ^, g/dL	BCS_H_/C_60_	33.8	33.7	34.3	34.6	34.5	35.2	35.3	34.8	0.2	<0.001	0.269	0.073	0.529	0.632	0.576	0.012
	BCS_H_/C_35_	33.9	34.0	34.5	34.8	34.6	34.5	35.1	35.1								
	BCS_A_/C_60_	34.4	34.2	34.5	34.8	35.1	34.6	34.8	35.0								
	BCS_A_/C_35_	33.8	34.1	34.8	34.9	35.1	35.0	35.1	34.6								
RDW ^◊^, %	BCS_H_/C_60_	19.65	19.35	18.59	18.38	18.28	18.17	17.52	17.33	0.55	<0.001	0.871	0.899	0.290	0.056	0.951	0.834
	BCS_H_/C_35_	18.31	18.33	17.88	17.63	17.70	17.41	17.26	17.08								
	BCS_A_/C_60_	19.53	19.28	18.71	18.73	18.27	17.85	17.56	17.49								
	BCS_A_/C_35_	18.98	18.45	18.15	18.04	17.75	17.36	17.20	17.04								

^§^ Before calving cows were assigned to high and adequate body condition score (BCS)-groups (BCS_H_, BCS_A_). ^$^ After calving both groups were subdivided again, each into a group with 35% concentrate feed proportion (C_35_) and a group with 60% concentrate feed proportion (C_60_) in the ration (increasing from 35–60% during the first three weeks after parturition). Thus, four groups emerged: BCS_H_/C_60_ (*n* = 15), BCS_H_/C_35_ (*n* = 15), BCS_A_/C_60_ (*n* = 15), BCS_A_/C_35_ (*n* = 15). ^$^ Mean corpuscular volume. ^¥^ Mean corpuscular hemoglobin. ^ǁ^ Mean corpuscular hemoglobin concentration. ^◊^ Red cell distribution width. ^+^ Values are presented as LSMeans. ^#^ Pooled standard error of means.
